# Prediction of Pilot's Reaction Time Based on EEG Signals

**DOI:** 10.3389/fninf.2020.00006

**Published:** 2020-02-14

**Authors:** Bartosz Binias, Dariusz Myszor, Henryk Palus, Krzysztof A. Cyran

**Affiliations:** ^1^Department of Data Mining and Engineering, Faculty of Automatic Control, Electronics and Computer Science, Silesian University of Technology, Gliwice, Poland; ^2^Department of Algorithmics and Software, Faculty of Automatic Control, Electronics and Computer Science, Silesian University of Technology, Gliwice, Poland; ^3^Department of Computer Vision Graphics and Digital Systems, Faculty of Automatic Control, Electronics and Computer Science, Silesian University of Technology, Gliwice, Poland

**Keywords:** aircraft control human factors, cognitive workload, data mining, electroencephalography, reaction time, safety, regression, prediction

## Abstract

The main hypothesis of this work is that the time of delay in reaction to an unexpected event can be predicted on the basis of the brain activity recorded prior to that event. Such mental activity can be represented by electroencephalographic data. To test this hypothesis, we conducted a novel experiment involving 19 participants that took part in a 2-h long session of simulated aircraft flights. An EEG signal processing pipeline is proposed that consists of signal preprocessing, extracting bandpass features, and using regression to predict the reaction times. The prediction algorithms that are used in this study are the Least Absolute Shrinkage Operator and its Least Angle Regression modification, as well as Kernel Ridge and Radial Basis Support Vector Machine regression. The average Mean Absolute Error obtained across the 19 subjects was 114 ms. The present study demonstrates, for the first time, that it is possible to predict reaction times on the basis of EEG data. The presented solution can serve as a foundation for a system that can, in the future, increase the safety of air traffic.

## 1. Introduction

Safety is an important consideration in the modern airline industry. Although many factors have an influence on the proper execution of flight processes, performance of the pilot is one of the most crucial factors. In particular, multiple sources point out that fatigue has a significant adverse impact on pilot performance (Lee and Kim, 2018; Bushmaker et al., 2019). The International Civil Aviation Organization (2016) defines fatigue as:

*A physiological state of reduced mental or physical performance capability resulting from sleep loss or extended wakefulness, circadian phase, or workload (mental and/or physical activity) that can impair a crew member's alertness and ability to safely operate an aircraft or perform safety related duties*.

Results of a survey published in 2002 demonstrate that fatigue is a significant issue among pilots, and may be responsible for 4–8% of aviation mishaps (Caldwell and Gilreath, 2002). Moreover, a survey conducted in a group of short-haul pilots points out that over 75% of pilots claimed that they have experienced significant fatigue (Jackson and Earl, 2006). In addition, over 70% of corporate pilots claimed that they have fallen into micro-sleep during various phases of the flight (Caldwell, 2005). Such micro-sleep states have been related to a reduced ability to respond to external stimuli (Ogilvie and Simons, 1992), as well as, degradation of performance on cognitive tasks (Belyavin and Wright, 1987).

Another large-scale study blames errors of the cockpit crew on 73% of the 456 aircraft crashes between the years 1959 and 1996 (National Research Council, 1998). Importantly, this trend does not seem to decrease over time, as the same source suggests that 72% out of the 145 accidents between the years 1987 and 1996 can be attributed to the cockpit crew.

In 2003, the National Transportation Safety Board estimated that fatigue contributes to around 20–30% of transportation accidents (i.e., aircraft, marine, railway, road). Given that ~70% of commercial aircraft accidents can be attributed to human errors, fatigue is thought to contribute to 15–20% of total aircraft accidents (Akerstedt et al., 2003).

Recent research study (Bennett, 2019) demonstrate that, on average, 7.3% of pilots who participated in this study and completed the inbound Top-of-Climb-Top-of-Descent scale were found to be either extremely tired or completely exhausted. In addition, 9.3% of pilots who completed the inbound Top-of-Descent-On-Blocks scale also claimed to be either extremely tired or completely exhausted. Of note, the Top-of-Climb-Top-of-Descent and Top-of-Descent-On-Blocks are phases of a flight. According to Bennet, these numbers could be even higher because there is a rule that pilots should not operate when fatigued; thus some pilots may under-report their fatigue level to avoid penalty. Exhaustion has been found to increase with the time of flight and Powell et al. estimated a linear relationship between tiredness and length of duty (Powell et al., 2007). It is worth mentioning that problems related to the workload and fatigue among pilots are important topics that have sparked recent changes in laws. For example, the European Aviation Safety Agency introduced new Flight Time Limitations (European Union Regulation 83/2014).

Considering the substantial impact of human factors on flight safety, there has been a rise of ideas and support for so-called, pilotless aircraft, in recent years (Ross, 2011; Stevenson, 2017). An approach that is most commonly postulated in this area is the idea of ground-based human or artificial intelligence support for a single pilot in an aircraft. However, a reduction in a number of on-board pilots might not necessarily be the best option, because the redundancy and support that two pilots provide to each other may be extremely valuable. Therefore, instead of removing pilots from cockpits, a more promising solution may be to support pilots with systems that can increase their capabilities and improve their performance during flights. The main hypothesis that will be tested in this work is that the electroencephalographic (EEG) signals recorded from a pilot's scalp during flight can be used in such performance-enhancing systems. In particular, we will test for associations between mental activity of pilots (as measured by EEG) and their ability to react quickly and make correct decisions in face of unexpected events. In this study, we also propose and test a basic pipeline that can be used for processing such signals and extracting information that can be used to predict a pilot's delay in response to unexpected events.

Use of EEG data in the context of prediction is most commonly associated with a seizure detection (Varsavsky et al., 2016). In a prospective study of antiepileptic drug withdrawal, a step-wise logistic regression analysis method was employed to predict an outcome of either antiepileptic drug withdrawal or seizure relapse (Overweg et al., 1987). However, an evaluation of the multivariate model showed that none of the variables that were related to the EEG signal contributed to the final score. A recent study presents a use of Deep Convolutional Neural Networks (CNN) for the automated detection and diagnosis of seizures using EEG signals (Acharya et al., 2018). Although CNN-based models are characterized by a high level of complexity, the additional preprocessing used in the work was limited to data standardization and normalization, and is thus fairly basic. Additionally, the aforementioned problem can be considered as more of a classification problem than a regression. EEG has already been utilized to predict a single-trial reaction time in a hand motor task (Meinel et al., 2015). The study by Meinel et al. used EEG band power features that were enhanced by a spatial filtering method called Source Power Comodulation. Alpha band power was found to comodulate with reaction time measured during an isometric hand force control task, which allowed for an average correlation of 0.19, with the best feature explaining up to 17% of the variation between single trials. Multiple studies have been performed to examine the impact of mental activity—as measured by EEG—on traffic safety. Most of these studies have been focused on car transport and driving. For instance, Deep Belief Networks (DBN) have been evaluated for their potential use in feature extraction and dimension reduction in predicting the cognitive state of drivers (Hajinoroozi et al., 2015). These studies show that DBN can predict around 85% of the variation in cognitive state. A subject-transfer framework for detecting drowsiness during simulated driving task based on EEG was also recently developed (Wei et al., 2018). In that study, response time was measured from the onset of a lane deviation to the onset of the response, which served as a behavioral assessment of drowsiness during the lane-keeping task. One interesting study associates periods of mind wandering during 20-min driving sessions with increased power in the alpha band of the EEG recording, as well as, a reduction in the magnitude of the P3a component of the event related potential in response to an auditory probe (Baldwin et al., 2017). Thus, these results suggest that, mind wandering can be detected on the basis of underlying brain physiology which has an impact on driving performance and the associated change in the driver's attentional state. Prior studies have documented changes in EEG activity that are present during the transition from normal drive to high mental workload and ultimately mental fatigue and drowsiness (Borghini et al., 2014). A review of the literature suggests that a high mental workload can be associated with increased EEG power in the theta band and a decreased power in alpha band. Additionally, increased EEG power in the theta, as well as, delta and alpha bands can be observed during the transition between mental workload and mental fatigue. Relatively fewer studies have explored the application of EEG data for the purpose of enhancing aircraft operations (Borghini et al., 2014). A recent study presented the idea of utilizing EEG signals in systems designed to monitor and enhance the performance of aircraft pilots (Binias et al., 2018). This work focuses on the problem of discriminating between states of brain activity related to idle but focused anticipation of a visual cue and the response to this cue. In this study, almost 78% average classification accuracy was obtained. This study can be regarded as a preamble to the work presented in the present article. Accordingly, to the best of our knowledge, no articles published to-date address the problem of predicting the delay in response time based on EEG activity. Therefore, the ideas presented in this article can be considered to be innovative and novel. In addition, the present study used simulators of the Virtual Flight Laboratory; thus, the experimental design used in this study is air-craft oriented. This design is valuable, as it targets a very important, yet not sufficiently explored field.

The remainder of this article is organized as follows. First, we provide a description of the experimental set-up and experimental protocol in section 2.1. Then, a steps of the EEG data processing pipeline proposed in this research are described in detail in section 2.3. Section 2.3.1 provides an overview of the tuning procedure used to find the optimal settings of prediction algorithms, and contains details about the algorithm validation procedure. The obtained results are presented in section 3. A general discussion about the results and the implemented approach can be found in section 4. Appendix A presents a brief theoretical background to all machine learning and statistical methods used in this work.

## 2. Materials and Methods

### 2.1. Study Population and Experiment Description

The goal of this experiment was to obtain the brain's bioelectrical activity prior to the occurrence of a visual cue. Additionally, we measured the time of delay in the participant's reaction time to that visual cue. To this end, we performed a series of experimental sessions. Each session consisted of a 2-h long simulated flight with activated auto pilot. Participants in this experiment were instructed to stay focused and maintain awareness while waiting for the appearance of the visual cue. Once the cue was observed, participants were instructed to press the button as quickly as possible. The location of the button was chosen to minimize the time required to react to the visual cue by restraining any additional movements of the pilots body, besides their fingers. Additionally, participants were asked to behave as pilots during regular flight, i.e., to observe cockpit instruments and scan the surroundings of the plane. The experiments took place in the Flight Navigational Procedure Training II class simulator and portrayed a *Cessna 172RG* airplane. To maintain consistency between successive experimental sessions, the simulated flight was on the route between Frankfurt and London. The same section of the flight was presented to each participant during the experiment. Flights took place at an average altitude of 6000 ft., and to simulate flight with auto pilot activated, the take off and landing were removed from the registered material. Moreover, the entire flight that was presented to participants took place over land. Importantly, sounds of engines were also generated in the cockpit.

Visual cues were displayed randomly with a normal distribution characterized by mean μ = 2.5 min, standard deviation σ = 1 min. This variance was introduced to prevent habituation of the human brain to regular patterns. The visual cue was represented by a solid gray-colored box that overlapped 75% of the main simulator screen that was responsible for displaying the terrain. Participants were between the ages of 20 and 65 years. Before start of the session, participants were asked to complete a survey regarding the level of their fatigue. All participants claimed that they were well rested before the session and all provided consent to utilize the outcomes obtained of the experiment for scientific research. During the experimentation phase, 19 participants (3 females and 16 males) were examined. Every experimental session started at the same time of the day—around 12:00 (noon)—to minimize the potential effects of external factors on the experiment.

This experiment was approved by the The Jerzy Kukuczka Academy of Physical Education in Katowice Bioethical committee (protocol number 2/1/2017).

### 2.2. Hardware Description

**Figure 1:**
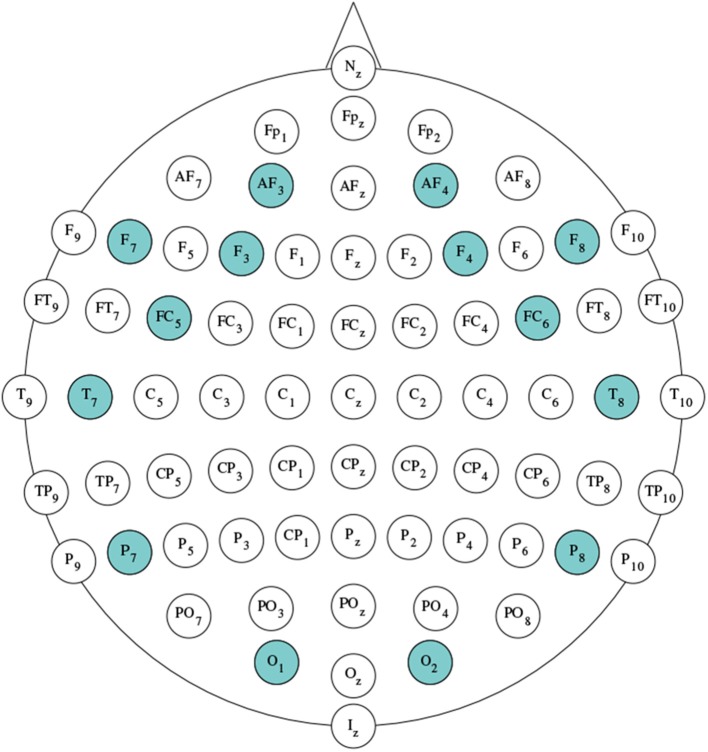
Positions of electrodes in the standard 10-10 electrode montage system. Highlighted locations reflect positioning of the Emotiv Epoc+ electrode with respect to 10-10 system-based on Koessler et al. (2009).

This study analyzed EEG signals to examine bioelectrical activity of participants' brains during the experiments. EEG signals were recorded using the Emotiv EPOC+ Headset. This device provides a useful bandwidth in the range of 0.16–43 Hz, and is sequentially sampled at a frequency of 128 Hz. The resolution of the data is on the level of 14 bit (1*LSB* = 0.51 μ*V*). To avoid interference of the electrical network, a real-time, digital 5-th order Sinc filter and notch filters at 50 and 60 Hz were built into *EPOC+* (EMOTIV Systems, 2014). The placement of EPOC+ electrodes follows the 10−10 configuration. Available channels are: AF3, F7, F3, FC5, T7, P7, O1, O2, P8, T8, FC6, F4, F8, AF4. with references in the P3/P4 locations. Emotiv headsets use active electrode placed in P3 location as an absolute voltage reference i.e., Common Mode Sense. The passive electrode located in P4 position serves as a feedback cancellation system to float the reference level on the common mode body potential i.e., Driven Right Leg (EMOTIV Systems, 2014). The position of electrodes is presented in Figure 1 (Koessler et al., 2009).

Emotiv EPOC+ is a relatively inexpensive EEG recording device that was designed for scientific research and other non-medical applications. Due to it's many advantages, EPOC+ is regularly used in Brain-Computer Interface (BCI) and similar solutions (Alrajhi et al., 2017; Setiono et al., 2018; Borisov et al., 2019). EPOC+ has also proven to be useful in a study concerning the classification of brain activity of pilots (Binias et al., 2018). A study evaluating EPOC+ in tasks that measured alpha brain activity and the Visual Steady-State Response showed that EPOC+ is capable of performing at levels comparable to research-grade EEG systems (Grummett et al., 2015). Due to setup difficulties, however, the authors of that study were unable to provide evidence to support the use of Emotiv in paradigms that rely on time-locked events. However, some reports of Emotiv EEG systems use in such tasks are available (Tahmasebzadeh et al., 2013).

### 2.3. Data Processing and Analysis

#### 2.3.1. Prediction of Response Delay

**Figure 2:**
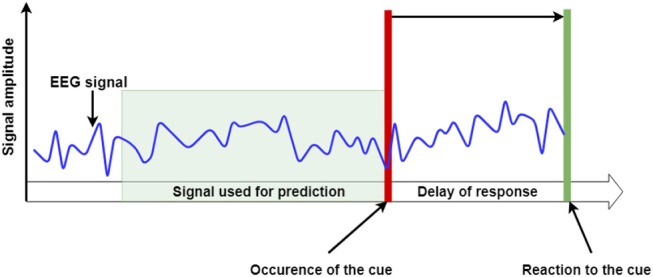
An illustrative representation of the EEG signal's TSI. The delay of response is calculated as the offset between the moment in time when the cue was presented to the subject and the moment when the subject's reaction to that cue was recorded. The prediction was made using only the segments of recorded EEG signal that immediately preceded the cue onset- or the “Temporal Segment of Interest” (TSI).

First, regression models were created to predict the delay in participant's response to the visual cue. The response delay is calculated as the offset between the moment in time when the cue was presented to the subject and the moment when subject's reaction to that cue was recorded. The prediction was made using only the segments of the recorded multichannel EEG signal that immediately preceded the onset of the cue. Such defined EEG segments will be referred to as the Temporal Segment of Interest (TSI). In particular, the length of the TSI is defined as the number of samples that will be considered when predicting the length of used time window. An illustrative representation of the concept of the TSI in the EEG signal and other defined names is presented in Figure 2.

**Figure 3:**
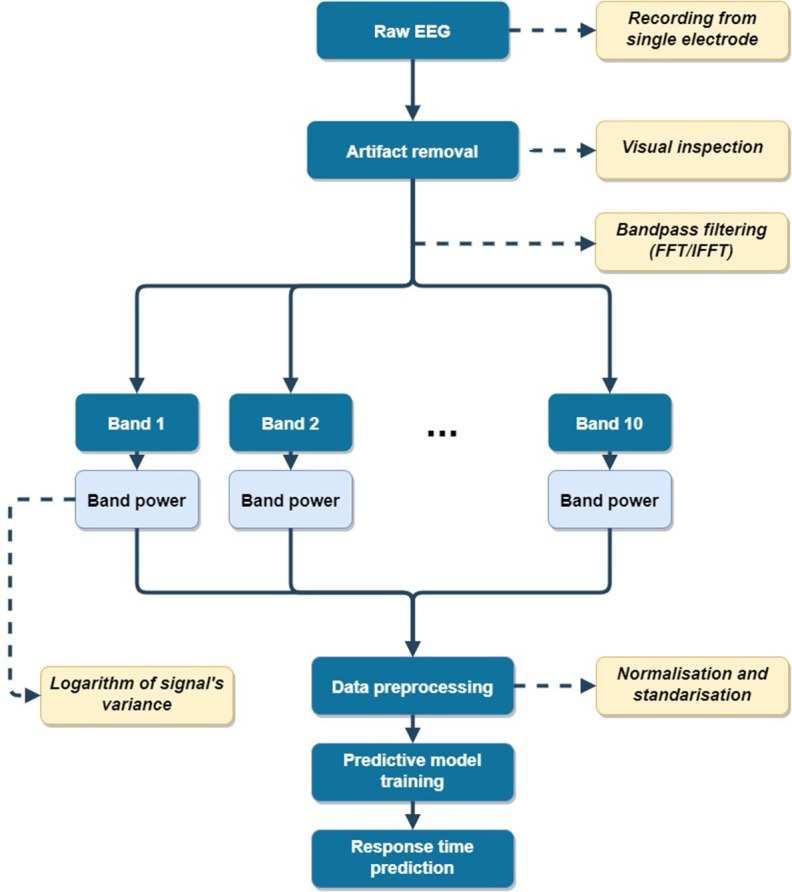
EEG signal processing pipeline (for single electrode).

Analysis of the raw, unprocessed signals in the TSI would not prove to be effective. Therefore, such data has to be appropriately preprocessed. First, the raw data were carefully examined to evaluate the significance of artifacts present in the recordings. A detailed description of this phase can be found in section 2.3.2. Next, the raw data from the TSI were divided into multiple signals on the basis of their frequency range, as described in section 2.3.3. From these signals, features were subsequently extracted according to procedure described in section 2.3.4. These features were used to train machine learning algorithms to predict the measured delay in a given subject's response to the occurrence of the visual cue. In the proposed approach, signal from each electrode is analyzed individually. A general flow of the EEG processing pipeline is presented in Figure 3.

Performance of machine learning models is dependent on the values of the variables-or “hyperparameters.” These hyperparameters differ based on different methods. The problem of choosing the optimal hyperparameters for a learning algorithm that minimizes a predefined loss function is called, hyperparameter optimization or tuning. For hyperparameter optimization, the present study used the Grid Search method (Bergstra et al., 2011). This approach involves an exhaustive searching through a manually specified subset of the hyperparameter space of a learning algorithm. Performance of various hyperparameter combinations was measured by 3-fold cross-validation on the training set with *Mean Absolute Error* (MAE) selected as the optimized performance metric.

For each subject, ~48 events were obtained during the experimental stage. Samples were then randomly divided so that 75% of samples were used for the training and tuning of prediction algorithms. The remaining 25% of the samples served as an independent dataset on which the best model (i.e., selected after hyperparameter optimization) was tested for each compared algorithm. To reduce the impact of random data division on the final score, datasets for each individual participant were randomly split into train-test datasets 11 times. *MAE* values obtained for each random repetition were then averaged for each subject. Let us assume that *y*_*m*_ is the real time of response delay for sample *m*, and the predicted delay response time for that sample is ŷ_*m*_. If *M* denotes the number of samples in the training set, then the final *MAE* value obtained from 11 cross-validations for subject *s* can be calculated with the following formula:

(1) $$\begin{array}{l} MAE_{s}=\frac{1}{11}\overset{11}{\underset{i=1}{\sum }}(\overset{M}{\underset{m=1}{\sum }}\frac{\left|{ŷ}_{m}-y_{m}\right|}{M}) \end{array}$$

A brief description of regression algorithms selected for the comparison can be found in Appendix A. A list of used hyperparameters and the searched space of their values for each algorithm is presented in Appendix B. For a detailed description of all hyperparameters, please refer to the documentation for the Python-based machine learning library *scikit-learn* (Pedregosa et al., 2011).

#### 2.3.2. Correction and Removal of Ocular Artifacts

Raw, multichannel time series data was obtained from EPOC+ devices during the experiment. Bioelectrical recordings from the brain are often contaminated with artifacts caused by muscle tensions, which are primarily related to eye movements and facial expressions. Given that these artifacts have a frequency spectrum that overlaps with part of the EEG spectra, the analysis of those signals is not only less effective, but in many cases, is impossible in their presence (Binias et al., 2015). Many approaches for filtering out artifacts and retrieving the underlying neural information have been proposed. Most commonly, regression methods are performed either in time or frequency domains (Binias et al., 2015). These artifact regression methods have been found to be highly effective. However, a requirement of providing at least one signal with a noise reference is a downfall for solutions that favor a limited number of electrodes in the configuration. This is particularly problematic for solutions that are designed for use in aircraft, which is the case for the system developed for the present study. On the other hand, if artifact regression is applied in the time domain, methods based on Adaptive Noise Cancelling (ANC) can be implemented for real-time applications. Indeed, this is a benefit of ANC approaches. There are various other techniques that can be used for detecting and filtering muscle movement-related artifacts, including blind source separation based algorithms (Jung et al., 2000). These algorithms include Principal Component Analysis (PCA) and Independent Component Analysis (ICA), which rely on recorded EEG and noise signals for calibration (Makeig et al., 1996). PCA and ICA approaches are particularly effective when a large amount of data is recorded across many channels. Similar to ANC-based approaches, the high data dimensionality requirement forces an extended electrode set-up, which is an inconvenience for practical solutions. Additionally, it must be noted that these methods function best in semi-automatic approaches, where supervision of an experienced user (i.e., expert) is required (Makeig et al., 1996). Although there are many eye blink correction and filtering methods described in the literature, proper validation of these methods a very demanding matter. To address this would require an uncontaminated EEG signal that can be used to compare the obtained corrected data, to evaluate the quality of filtering. However, since EEG signals are recorded with disturbances already additively mixed, there is no precise way to extract an original, desired component. Thus, it is impossible to recover the exact morphology of the uncontaminated signal and consequently, no unambiguous way of evaluating the accuracy of the reconstruction of the filtered signal (Binias and Niezabitowski, 2017). In light of these limitations, we decided to simply remove highly contaminated TSIs from further analysis. This approach is commonly used in clinical practice. EEG segments were therefore visually inspected for the presence of artifacts that had an amplitude multiple times greater than that of the surrounding data. Based on this criteria, careful inspection of the data revealed that no EEG segments were removed due to their contamination. Since the main goal of this work was to provide an initial validation of the stated thesis rather than to propose a production ready solution, an automatic artifact removal method was not necessary. Additional motivation behind this approach was that the solution described in this work should serve as a baseline and reference for future improvements.

#### 2.3.3. Frequency Analysis

As developments in neuroscience suggest, neural oscillations and their synchronization represent important mechanisms for inter-neuronal communication and the binding of information processed in distributed brain regions (Roach and Mathalon, 2008). Therefore, EEG signals are often analyzed based on their frequency characteristics. Indeed, time-frequency analysis of EEG signals can provide information on which frequencies have the most power at specific points in time and in certain location in the cortex. In the present study, the samples preceding the occurrence of the visual cue i.e., the TSI, will represent neural activity in the moment when performing of an action is required. The information about the spatial nature of observed processes will be obtained from the location of the EEG electrodes. In the proposed pipeline, EEG signals are analyzed in the following frequency bands, which correspond to specific brainwaves (Nunez and Srinivasan, 2006):

- Delta (1–4 Hz) (Landolt et al., 1996; Amzica and Steriade, 1998),
- Theta (4–8 Hz) (Strijkstra et al., 2003),
- Alpha (8–12 Hz) (Beatty, 1971; Strijkstra et al., 2003),
- Low Beta (12–16 Hz) (Beatty, 1971; Ang et al., 2012),
- Middle Beta (16–20 Hz) (Beatty, 1971; Ang et al., 2012),
- Middle-High Beta (20–24 Hz) (Beatty, 1971; Ang et al., 2012),
- High Beta (24–28 Hz) (Beatty, 1971; Ang et al., 2012),
- Gamma 1 (32–36 Hz) (Teplan, 2002; Ang et al., 2012),
- Gamma 2 (36–40 Hz) (Teplan, 2002; Ang et al., 2012),
- Broad band range (8–30 Hz) that is commonly related to the planning of motor movement (Blankertz et al., 2008).

Such bands have proven to be highly useful in a recent study that focused primarily on the problem of EEG-based discrimination between states of brain activity related to idle but focused anticipation of a visual cue and the response to that cue (Binias et al., 2018).

Since EEG is traditionally modeled as a series of sine waves of different frequencies that overlap in time and have different phase angles, the use of Fast Fourier Transform (FFT) for the frequency decomposition of such signal seems to be the most intuitive approach. To obtain bandpass filtered subsignals, each TSI was first decomposed into frequency components using FFT, for each channel separately. Then, the undesired frequencies were removed by changing their Fourier amplitudes to 0. Finally, the filtered signal was reconstructed using this modified Fourier representation using Inverse Fourier Transform algorithm. Although a detailed description of FFT is beyond the scope of this article, one important aspect of this approach warrants mention. That is, it is widely accepted that the larger the length of time window used for the FFT, the greater the frequency resolution of analysis. However, increasing the length of the TSI comes at the cost of decreasing the temporal resolution. This decrease in temporal resolution might cause a situation where the analyzed signal no longer represents the bioelectrical state of a subject's brain prior to the action requirement. As a result, these data might not be useful for predicting the delay in response. This problem is captured in the Heisenberg uncertainty principle (Folland and Sitaram, 1997). To address this problem, the present study utilized the, zero-padding, approach (Marple and Marple, 1987). In this method, the analyzed segment of a signal is extended by a sequence of zeros. This extended sequence is represented as a low frequency peak in the Fourier amplitude spectrum. If such addition is correctly treated during the analysis (i.e., discarded), it won't negatively affect the outcome, but it will increase the frequency resolution. Given that frequency components lower than 1 Hz are not considered in the present study, the zero padding approach could be implemented. For the purpose of this research, 0.5 s time windows were used, which corresponds to 64 samples of TSI length. Analyzed segments were additionally padded with 192 zeros so that the total length of signal to be decomposed with FFT was 256 samples.

#### 2.3.4. Feature Extraction

A common assumption is that changes in EEG power reflect changes in underlying neuronal activity (Roach and Mathalon, 2008). These power changes are typically referred to as Event-Related Synchronization and Event-Related Desynchronization, to describe the changes in EEG power that are related to the occurrence of a specific event (Pfurtscheller and Da Silva, 1999). Therefore, one of the most effective and widely used descriptors of EEG data is the power of the signal calculated in a specific frequency range (Blankertz et al., 2008). Since the mean value of the bandpass filtered signal tends to zero, the variance of such signal can be used to represent its bandpower. To improve the performance of chosen classification algorithm, the distribution of the extracted bandpower features is often normalized using a natural logarithm function (Binias et al., 2016a). The logarithm of variance feature, that will also be referred to as *logvar*, was chosen as the descriptive statistics in the described pipeline. Since the experimental set up consists of 14 electrodes and each signal is further decomposed into 10 frequency subbands, a total of 140 logvar features were obtained for each trial i.e., appearance of visual cue, in each experiment. Before tuning and training of the prediction algorithms, all features were subjected to the classical standardization and normalization procedures to obtain a zero mean value and unitary standard deviation. Section 2.3.1 contains a detailed description of the implemented approach to the problem of regression.

## 3. Results

Summary statistics for delay times in response to the cue and a total number of epochs registered for each subject, are presented in Table 1. One of the initial hypotheses was that the delay in reaction time will increase with an increase in the duration of the experiment. To determine whether a relation between the time in experiment when the event happened and response delay, a Robust Linear Model (RLM) was fit to the data. The RLM is estimated via iteratively reweighted least squares (Huber, 1973). The robust criterion function used for downweighting the outliers was Hubers T for M estimation (Huber, 1973; Huber et al., 2013). A more detailed description of this approach lies beyond the scope of this article. The explanatory variable used for the modeling was the timestamp of the event i.e., cue appearance. The delay in response time was the explained variable. Table 1 shows observed slope coefficients of fitted lines, as well as, *p*-values describing their statistical significance. Only for subjects 6, 7, 8, 9, 13, 14, 16, and 18, *p*-values of the slope coefficients were lower than 0.03 and can therefore be considered statistically significant. Slope coefficients for those subjects, as well as for other subjects, are very close to 0. Given these observations, it can be assumed that neither a linearly increasing nor decreasing trend can be attributed to the changes in response delay over time. Further analysis was conducted on the basic statistics of the data presented in Table 1, especially the standard deviation σ and the difference between minimal and maximal values for each subject with respect to the median. These additional analyses suggest high variability in response time values throughout each session.

**Table 1:** Basic statistics of the response delay times summarized for each subject.

**Subject**	**Min. [s]**	**Median [s]**	**Max. [s]**	**σ [s]**	**Slope [s/s]**	***p*-value**	**No. epochs**
1	0.408	0.553	1.616	0.305	1.82E-12	0.161	50
2	0.468	0.659	1.129	0.154	7.26E-13	0.527	44
3	0.402	0.592	1.124	0.163	–3.61E-12	0.120	46
4	0.450	0.627	0.934	0.130	–2.35E-12	0.393	45
5	0.386	0.495	0.784	0.097	1.01E-12	0.106	50
6	0.304	0.408	0.704	0.087	–1.14E-12	0.016	48
7	0.443	0.616	1.498	0.234	2.76E-12	0.027	49
8	0.323	0.544	2.113	0.426	7.43E-12	0.020	48
9	0.381	0.487	1.086	0.128	2.26E-12	<1E-5	50
10	0.658	1.199	3.279	0.613	6.57E-12	0.468	48
11	0.412	0.541	0.743	0.093	–8.59E-13	0.250	47
12	0.421	0.560	0.994	0.129	–7.99E-13	0.208	49
13	0.414	0.759	2.610	0.432	5.47E-12	<1E-5	51
14	0.379	0.664	1.982	0.238	–1.90E-12	0.025	49
15	0.278	0.421	0.665	0.091	3.90E-14	0.956	47
16	0.436	1.009	3.192	0.749	1.48E-11	<1E-5	43
17	0.390	0.555	1.660	0.229	–8.87E-13	0.301	50
18	0.375	0.547	1.296	0.181	1.59E-12	0.017	53
19	0.362	0.533	1.947	0.297	–1.99E-12	0.078	51

Average *MAE* scores obtained for different prediction algorithms are presented in Table 2. It can be observed that the best average results were obtained with the SVMRBF algorithm (114 ms). What is worth to notice is that MAE for subjects 10 and 16 is much higher than that of other subjects. However considering that the average reaction delay was around 600 ms, this is a relatively small error. Therefore, the obtained results can be considered satisfactory. Additionally, the standard deviations of absolute errors (AE) were taken into account and presented in Table 3. Again, the SVMRBF results were characterized by the lowest value of 68 ms. The maximal prediction AEs are shown in Table 4. Given that the presented solution is meant to be utilized for safety solutions in the future, this metric is especially important. Failing to predict a single decrease in performance (i.e., a drastic increase in response delay) might lead to more serious consequences than averaging a relatively higher mean error for all events. The average maximal prediction absolute error exceeded 200 ms for all algorithms, with SVMRBF outscoring other algorithms by at least 24 ms.

**Table 2:** Comparison of prediction's Mean Absolute Errors obtained for each subject.

**ID**	**LASSO [s]**	**LASSO-LARS [s]**	**KernelRidge [s]**	**SVMRBF [s]**	**Shuffled SVM [s]**
1	0.140	0.140	0.144	0.125	0.230
2	0.077	0.077	0.082	0.082	0.144
3	0.076	0.070	0.062	0.077	0.138
4	0.081	0.081	0.103	0.092	0.164
5	0.062	0.062	0.059	0.073	0.091
6	0.046	0.046	0.044	0.050	0.066
7	0.121	0.121	0.138	0.121	0.196
8	0.264	0.264	0.210	0.231	0.317
9	0.067	0.066	0.066	0.065	0.086
10	0.292	0.292	0.380	0.265	0.425
11	0.063	0.063	0.063	0.064	0.079
12	0.058	0.058	0.053	0.061	0.088
13	0.190	0.190	0.199	0.132	0.233
14	0.085	0.086	0.094	0.069	0.104
15	0.058	0.058	0.050	0.059	0.069
16	0.411	0.409	0.406	0.340	0.684
17	0.105	0.105	0.138	0.087	0.146
18	0.074	0.074	0.076	0.073	0.127
19	0.105	0.105	0.106	0.108	0.167
AVG	0.125	0.125	0.130	0.114	0.187

**Table 3:** Comparison of Absolute Errors Standard Deviations obtained for each subject.

**ID**	**LASSO [s]**	**LASSO-LARS [s]**	**KernelRidge [s]**	**SVMRBF [s]**	**Shuffled SVM [s]**
1	0.103	0.103	0.107	0.082	0.170
2	0.062	0.062	0.073	0.056	0.102
3	0.039	0.037	0.034	0.044	0.085
4	0.056	0.056	0.072	0.061	0.102
5	0.044	0.044	0.040	0.046	0.053
6	0.020	0.020	0.024	0.025	0.038
7	0.066	0.066	0.081	0.076	0.163
8	0.141	0.142	0.123	0.163	0.262
9	0.038	0.037	0.040	0.038	0.052
10	0.165	0.165	0.196	0.111	0.230
11	0.040	0.040	0.041	0.037	0.040
12	0.040	0.040	0.044	0.042	0.061
13	0.119	0.119	0.121	0.089	0.255
14	0.055	0.055	0.057	0.044	0.081
15	0.033	0.033	0.034	0.032	0.041
16	0.253	0.252	0.312	0.172	0.590
17	0.061	0.061	0.091	0.054	0.156
18	0.058	0.09	0.058	0.055	0.131
19	0.086	0.086	0.088	0.073	0.140
AVG	0.078	0.078	0.086	0.068	0.145

**Table 4:** Comparison of Maximal Absolute Errors obtained for each subject.

**ID**	**LASSO [s]**	**LASSO-LARS [s]**	**KernelRidge [s]**	**SVMRBF [s]**	**Shuffled SVM [s]**
1	0.305	0.304	0.313	0.261	0.476
2	0.180	0.180	0.213	0.168	0.311
3	0.119	0.113	0.103	0.132	0.240
4	0.144	0.144	0.188	0.164	0.282
5	0.129	0.129	0.120	0.143	0.172
6	0.076	0.076	0.083	0.090	0.129
7	0.211	0.211	0.262	0.236	0.485
8	0.446	0.448	0.380	0.467	0.706
9	0.122	0.119	0.124	0.120	0.169
10	0.505	0.505	0.628	0.399	0.719
11	0.131	0.131	0.132	0.126	0.140
12	0.124	0.124	0.131	0.129	0.188
13	0.389	0.389	0.398	0.284	0.719
14	0.167	0.171	0.176	0.139	0.249
15	0.110	0.110	0.103	0.115	0.136
16	0.814	0.911	0.873	0.605	1.666
17	0.201	0.201	0.274	0.161	0.439
18	0.168	0.176	0.177	0.168	0.390
19	0.250	0.250	0.257	0.243	0.442
AVG	0.242	0.242	0.260	0.218	0.424

On average, all scores of both LASSO-based algorithms and Kernel Ridge regression were off by a few milliseconds with respect to SVMRBF. In order to properly examine the performance differences between compared algorithms a one-way ANOVA test was performed, where all AEs of prediction were used as observations and each of the regression algorithms was representing an individual group. The computed *F*-value of one-way ANOVA test was 2.246. The associated *p*-value from the F-distribution was 0.081. Since the results of performed ANOVA tests indicate the existence of statistically significant, albeit subtle, differences between AE obtained within each group *post-hoc t*-tests were performed to investigate this furthermore. Table 5 presents *p*-values obtained from performed *t*-tests. The results indicate that the SVMRBF algorithm allowed to obtain a significantly (*p* < 0.05) values of AE.

**Table 5:** *p*-values of pairwise *t*-tests performed in order to compare absolute errors of prediction obtained with different regression algorithms.

***p-value***	**LASSO**	**LASSO-LARS**	**KernelRidge**	**SVMRBF**	**Shuffled SVM**
LASSO		0.991	0.627	0.043	<1E-7
LASSO-LARS	0.991		0.619	0.044	<1E-7
KernelRidge	0.627	0.619		0.014	<1E-6
SVMRBF	0.043	0.044	0.014		<1E-10
Shuffled SVM	<1E-7	<1E-7	<1E-6	<1E-10	

An additional analysis was carried out in order to validate the proposed solution further. For this purpose, the best performing algorithm—the SVMRBF—was trained with shuffled reaction times. The motivation behind that is to compare how well does the prediction work against simply learning to predict the average reaction time for each subject. Average MAEs obtained for each subject with this approach are presented in Table 2 under *Shuffled SVM* column. Insignificant differences in MAE between properly trained algorithms and this would indicate that proposed approach is not using EEG information. *F*-value of performed one-way ANOVA (with *Shuffled SVM* included as one of the groups) was 20.901 (associated *p*-value is less than 10^−16^). This indicates some statistical differences between groups and justifies performing additional *post-hoc t*-tests. Results presented in Table 5 prove that all proposed algorithms perform significantly better than fitting average.

**Figure 4:**
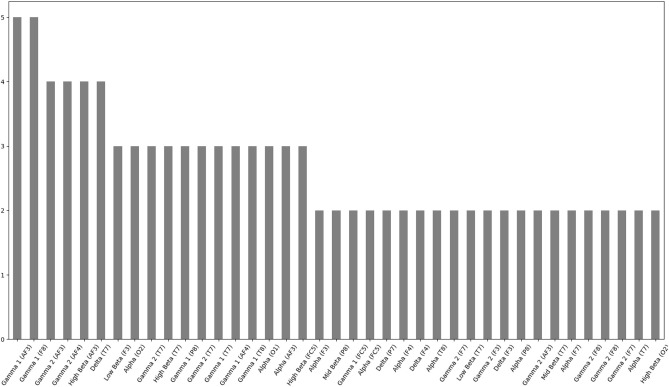
Histogram of cumulative feature selections for all subjects for SVMRBF algorithms. Only features selected more than once were included.

Since the best performing regression algorithm—SVMRBF—requires initial feature ranking and selection, analysis of the nature of top predictors could provide an interesting and valuable information. Presented in Table 6 is a summary of most commonly selected features, across all 11 cross-validation, for each individual subject. Figure 4 presents a histogram of top feature selections. It can be observed that optimal subset of features varies highly between subjects with most frequently selected features—Gamma 1 in AF3 electrode location and Gamma 1 in F8 electrode location—being common only for 5 subjects each. Further features—Gamma 2 (AF3 electrode), High Beta (AF3 electrode), Delta (T7 electrode) and Gamma 2 (AF4 electrode)—were common only for 4 subjects.

**Table 6:** Summary of top features selected most commonly for individual subjects, as well as, for all subjects combined for SVMRBF algorithm.

**ID**	**Top features**
1	Delta (T7), Gamma 1 (FC5), Gamma 2 (F7), Gamma 2 (FC5),Gamma 2 (F7), High Beta (FC5), High Beta (T7), Low Beta (T7), Broad (T7), Mid Beta (T7)
2	Alpha (AF3), Alpha (F4), Alpha (F7), Alpha (T8), Delta (T8), Gamma 1 (P8), High Beta (T8), Broad (F7), Broad (T8), Mid Beta (P8)
3	Alpha (F3), Gamma 2 (AF3), Gamma 2 (F3), Low Beta (F3), Mid Beta (P8)
4	Alpha (O1), Alpha (T7), Delta (AF4), Delta (F3), Delta (F4), Delta (F7), Delta (FC6), Delta (P7), Delta (T7), Low Beta (F3)
5	Delta (P7), Gamma 2 (P7),Gamma 2 (P7), Mid Beta (P7)
6	Gamma 1 (AF3), Broad (AF4), Broad (F8), Raw (AF3), Raw (AF4)
7	Alpha (O1), Alpha (O2), Gamma 1 (F8), Gamma 2 (AF4), Gamma 2 (F8), Mid Beta (F3)
8	Gamma 1 (AF3), Gamma 1 (AF4), Gamma 1 (F7), Gamma 1 (F8), Gamma 1 (T8), Gamma 2 (AF3), Gamma 2 (AF4), Gamma 2 (O2),Gamma 2 (F8),Gamma 2 (FC5)
9	Alpha (T7), Delta (T7), Gamma 2 (T7),Gamma 2 (T7), High Beta (T7), Low Beta (T7), Mid Beta (T7), Theta (T7)
10	Alpha (AF3), Alpha (F3), Alpha (F4), Alpha (F7), Alpha (F8), Alpha (FC5), Alpha (FC6), Alpha (T8),Gamma 2 (F3),Gamma 2 (F7)
11	Gamma 1 (P8), Gamma 2 (F7),Gamma 2 (T7), High Beta (FC5), Low Beta (F4), Low Beta (P8)
12	Alpha (P7), Delta (F3), Gamma 2 (AF3),Gamma 2 (F4),Gamma 2 (FC6), High Beta (AF3), High Beta (O2), High Beta (T7), Broad (P7), Raw (F3)
13	Gamma 1 (F8),Gamma 2 (F8), High Beta (F7), High Beta (F8), High Beta (O2), Raw (F4)
14	Alpha (AF3), Gamma 1 (AF3), Gamma 2 (AF3),Gamma 2 (AF3), High Beta (AF3), Low Beta (F3)
15	Alpha (P8), Delta (F4), Gamma 1 (AF4), Gamma 1 (F8), Gamma 1 (O1), Gamma 1 (P8), Gamma 1 (T8), Gamma 2 (AF4), Gamma 2 (F8), Gamma 2 (T8)
16	Alpha (FC5), Alpha (O1), Alpha (O2), Alpha (P8), Delta (T7), Gamma 1 (T7), Gamma 2 (T7), Gamma 2 (T7), High Beta (AF3), High Beta (FC5)
17	Gamma 1 (AF3),Gamma 2 (AF3), High Beta (AF3), Low Beta (AF3)
18	Gamma 1 (AF3), Gamma 1 (AF4), Gamma 1 (F3), Gamma 1 (F4), Gamma 1 (F8), Gamma 1 (FC5), Gamma 1 (T7), Gamma 1 (T8), Gamma 2 (AF4), Gamma 2 (F3)
19	Alpha (O2), Gamma 1 (T7), Gamma 2 (T7), Low Beta (AF4), Broad (O2)
Overall	Gamma 1 (AF3), Gamma 1 (F8), Gamma 2 (AF3), High Beta (AF3), Delta (T7), Gamma 2 (AF4)

*Frequency ranges correspond to those listed in section 2.3.3. Values in brackets correspond to electrode locations. Features are sorted alphabetically*.

## 4. Discussion

The present study provides a novel utilization of EEG to predict delays in response time. Indeed, we demonstrated that it is possible to obtain satisfactory results based solely on the processed EEG signals. The average MAE value for SVMRBF was 114 ms. This is a relatively small error, which indicates that the achieved results are very promising. This is particularly true given that this is the initial phase of this work and the first time that this problem has been approached. For all subjects, the MAE was at least a few times smaller than their lowest reaction delay. The other tested regression algorithms performed significantly worse than SVMRBF; however the gap between LASSO-LARS, LASSO, and Kernel Ridge regression was only couple seconds. With the lowest standard deviation of prediction's AE, SVMRBF was also the most precise algorithm. Additional tests proved that proposed solution performs significantly better than simple average fitting.

Errors obtained for 12 subjects did not exceed 100 ms. A higher score for some of the subjects emphasizes the complexity of the problem. Additionally, another important observation can be made—that none of the algorithms resulted in the lowest MAE for all subjects. Altogether, these findings might indicate that subject-specific algorithm selection might improve the performance of the proposed solution. However, the significantly higher errors for few subjects could be related to the phenomena known as BCI illiteracy (Allison and Neuper, 2010). Indeed, some studies suggest that there is a group of people not capable of using EEG-based BCI systems (Allison and Neuper, 2010; Vidaurre and Blankertz, 2010). While this possibility must be taken into consideration in future work this conclusion should not be drawn hastily to explain the poorer than expected performance of the proposed solution for some subjects.

The statistics presented in Table 1 suggest no significant trend i.e., neither increasing nor decreasing in the lengths of delay in response times. Additionally, high values of standard deviations (compared to the median) might indicate that the times are random, or at least independent from obvious variables such as timestamp of experiment. Such high variability in the data is a good prognostic indicator of the experiment. In particular, when designing machine learning algorithms, great care needs to be taken to avoid tuning the model to strong correlations that have no actual relation to the explained or explanatory variables. If the data where instead aligned to any monotonic function that is dependent upon the timestamp, then relatively low regression errors could be obtained; however, EEG-related variables would have a negligible impact on that score. Since that is not a case, the obtained results can be considered satisfactory with a greater confidence.

The analysis of selected features for—the most effective—SVMRBF algorithm was additionally performed. A high variability between the optimal subsets of features selected for individual subjects was observed. In particular, the greatest number of subjects for whom same features were common (Gamma 1 in AF3 electrode location and Gamma 1 in F8 electrode location) was 5. This is merely over 25% of the total number of subjects. Therefore, no detailed conclusions about the mental processes underlying fast reaction related actions can be drawn at this stage of the experiment. Such differences can be explained by both, or either of individual characteristics of neural activity related to the presented task or overlapping of bioelectrical source activity caused by the effects of volume conduction. It is a common knowledge that due to this phenomena analysis of cortical activity may be less precise. Additionally, some important spatiotemporal features of the EEG signal might not be properly observed (Blankertz et al., 2008). Therefore, among the most important future additions to the pipeline is the implementation of a spatial filtering step (Blankertz et al., 2008). The use of a spatial filtering algorithm has proven to be highly beneficial in various EEG bandpower-based solutions (Binias et al., 2016b, 2018). Authors believe that such addition would no only allow to further decrease the prediction MAE, but also make the analysis of most relevant frequency bandwidths and cortical locations more accurate and exhaustive.

Another feature that should be tested, that may have an impact on prediction error is the removal and correction of short-time, high-amplitude artifacts such as eye movement, blinking, and muscle activity. Several approaches, including Artifact Subspace Reconstruction (ASR) or rejecting the subspace of ICA coefficients, may provide a potent solution to this problem (Le et al., 2011; Akhtar et al., 2012; Mullen et al., 2013). Due to its capability for real-time application, the ASR method, in particular, should be considered for addition to the pipeline.

The presented solution may serve as a starting point for future concepts and improvements. The idea of predicting the delay in response time to an unexpected event hides a much broader concept than the one reflected in the present experiment. The constant monitoring of predicted reaction time might shed new light on how pilot's capabilities change over the course of a flight. These changes over time might then be used to trigger an alarm once a significant decrease in predicted reaction time is expected. Such an approach to addressing the problem would then provide an overview of the overall level of fatigue, rather than being a temporally-limited metric. A future follow-up experiment will be conducted that includes a larger sample size, and a measurement device that provides greater coverage of the brain's cortical areas. This followup experiment will validate the proposed approach and test the potential of the implemented solution.

## Data Availability Statement

The datasets generated for this study are available on request to the corresponding author.

## Ethics Statement

The studies involving human participants were reviewed and approved by The Jerzy Kukuczka Academy of Physical Education in Katowice Bioethical Committee. The patients/participants provided their written informed consent to participate in this study.

## Author Contributions

BB designed and implemented the entire digital signal processing pipeline and data analysis methodology. Additionally, BB performed all of the described data analyses and prepared this manuscript. All data was obtained by DM, who additionally designed, created, and/or configured all software used for the purpose of the EEG recording. DM has also provided substantive consultation regarding the applied and designed methodology, and contributed to introduction, study population and experiment description, and discussion of this manuscript. The original research idea and plan of the conducted experiment was designed equally by BB and DM. HP provided substantive consultation throughout the course of the experiment, particularly (but not limited to) with respect to the applied statistical and machine learning methods. Additionally, HP assisted with the editorial organization of the article's content. KC provided substantive consultation regarding the aspects of the research that are related to aircraft.

### Conflict of Interest

The authors declare that the research was conducted in the absence of any commercial or financial relationships that could be construed as a potential conflict of interest.
