# Supplementary material for: Prediction of Pilot's Reaction Time Based on EEG Signals
Source: Front Neuroinform. 2020 Feb 14;14:6. doi: 10.3389/fninf.2020.00006 (PMC7033428; doi:10.3389/fninf.2020.00006)
Supplement: Supplementary file 1 [file Presentation_1.pdf]

# Prediction of Pilot's Reaction Time Based on EEG Signals - Appendix

**Bartosz Binias** [1*], **Dariusz Myszor** [2], **Henryk Palus** [1] **and Krzysztof A. Cyran** [3]

[1] *Department of Data Mining and Engineering, Faculty of Automatic Control, Electronics and Computer Science, Silesian University of Technology, Gliwice, Poland*

[2] *Department of Department of Algorithmics and Software, Faculty of Automatic Control, Electronics and Computer Science, Silesian University of Technology, Gliwice, Poland*

[3] *Department of Computer Vision Graphics and Digital Systems, Faculty of Automatic Control, Electronics and Computer Science, Silesian University of Technology, Gliwice, Poland*

Correspondence*:
Bartosz Binias
bartbinias@gmail.com

## REFERENCES

Binias, B., Myszor, D., and Cyran, K. A. (2018). A machine learning approach to the detection of pilot's reaction to unexpected events based on EEG signals. *Computational Intelligence and Neuroscience* 2018

Binias, B., Palus, H., and Niezabitowski, M. (2016). Elimination of bioelectrical source overlapping effects from the EEG measurements. In *Carpathian Control Conference (ICCC), 2016 17th International* (IEEE), 70–75

Efron, B., Hastie, T., Johnstone, I., Tibshirani, R., et al. (2004). Least angle regression. *The Annals of Statistics* 32, 407–499

Friedman, J., Hastie, T., and Tibshirani, R. (2010). Regularization paths for generalized linear models via coordinate descent. *Journal of Statistical Software* 33, 1

Kraskov, A., Stögbauer, H., and Grassberger, P. (2004). Estimating mutual information. *Physical Review E* 69, 066138

Pedregosa, F., Varoquaux, G., Gramfort, A., Michel, V., Thirion, B., Grisel, O., et al. (2011). Scikit-learn: Machine Learning in Python. *Journal of Machine Learning Research* 12, 2825–2830

Robert, C. (2014). Machine learning, a probabilistic perspective. *CHANCE* 27, 62–63. doi:10.1080/09332480.2014.914768

Ross, B. C. (2014). Mutual information between discrete and continuous data sets. *PloS One* 9, e87357

Smola, A. J. and Schölkopf, B. (2004). A tutorial on Support Vector Regression. *Statistics and Computing* 14, 199–222

## A DESCRIPTION OF STATISTICAL AND MACHINE LEARNING METHODS

### A.1 Feature standardization and mean removal

Feature standardization and mean removal i.e., normalization, is a common practice in many machine learning approaches. The procedure is performed by removing the mean of the feature vector and scaling

it to unit variance. This procedure is then applied to each feature vector independently by computing the relevant statistics on the basis of samples from the training set. Mean and standard deviation are then stored for use on an independent test data.

If we denote the mean value of training samples from feature $f$ by $\mu(X_f)$ and their standard deviation as $\sigma(X_f)$, then for a sample $x_{fi}$ ($i = 1, \ldots, M_f$, $M_f$ - is a number of samples of feature $f$), the described transformation can be calculated using the following equation:

$$z_{fi} = \frac{x_{fi} - \mu(X_f)}{\sigma(X_f)} \tag{1}$$

In this research study $f$ denotes the bandpower features calculated from $10$ frequency bands defined in Sec. 2.3.3.

## A.2 Mutual Information

Mutual Information (MI) between two random variables is a non-negative value that describes the dependency between these variables. MI is equal to zero if and only if two random variables are independent. At the same time, higher values of the MI score indicate a higher dependency. If $X_f \in \mathbf{R}^{M_f}$ denotes the vector of $M_f$ samples of feature $f$ and $Y \in \mathbf{R}^{M_f}$ is a vector of corresponding targets, then the MI of these two discrete variables can be defined as in Eq. 2. In this study, MIs for discrete variables were obtained with nonparametric methods based on entropy estimation from k-nearest neighbors distances (Kraskov et al., 2004; Ross, 2014).

$$MI(X_f;Y) = \sum_{y \in Y} \sum_{x_f \in X_f} p(x_f, y) log\Big(\frac{p(x_f, y)}{p(x_f)p(y)}\Big) \tag{2}$$

In Eq. 2, $p(x_f, y)$ is the joint probability function of $X_f$ and $Y$, and $p(x_f)$ and $p(y)$ are the marginal probability distribution functions of $X_f$ and $Y$ respectively. In this study, $x_f$ is a vector of 10 bandpower features obtained for a single event, while $y$ is the corresponding time of delay in reaction to that event.

The MI criterion is known for being capable of capturing any kind of dependency between variables. Use of MI-based feature selection methods have been proven to yield highly satisfactory results in many approaches to EEG signal processing (Binias et al., 2016, 2018).

## A.3 F-regression

F-test statistics can be used as a criterion for ranking features. This approach utilizes univariate linear regression for testing the individual effect of the regression variables. To extract this information, the first step requires that the correlation between the vector of regressors $X_f \in \mathbf{R}^{M_f}$ and the vector of targets $Y \in \mathbf{R}^{M_f}$ is computed, according to the following equation:

$$R_f^2 = \frac{\big(X_f - \mu(X_f)\big)^T\big(Y - \mu(Y)\big)}{\sigma(X_f)\sigma(Y)} \tag{3}$$

The $R_f^2$ is then converted to an F-score to obtain the final result. If we denote the number of observations as $M_f$ and the degrees of freedom as $p_f$, then the relation between the F-score $F_f$ and $R_f^2$ is expressed as in Eq.4.

$$R_f^2 = 1 - \big(1 + F_f \frac{p_f - 1}{M_f - p_f}\big)^{-1} \tag{4}$$

It must be noted that the F-test expresses only a linear dependency between variables. In this study, $x_f$ is a vector of $10$ bandpower features obtained for a single event, while $y$ is the corresponding time of delay in reaction to that event.

## A.4 Least Absolute Shrinkage and Selection Operator

Least Absolute Shrinkage and Selection Operator (LASSO) is a linear model that estimates sparse coefficients. Mathematically, the optimization objective for trained linear model is the $L1$ norm regularizer defined by the following equation (Friedman et al., 2010):

$$L1 = \min_w \frac{1}{2N} \|Xw - Y\|_2^2 + \alpha \|w\|_1 \tag{5}$$

where:

- $X \in \mathbf{R}^{M \times N}$ - input data (bandpower features),
- $Y \in \mathbf{R}^M$ - target (vector of reaction times),
- $\|w\|_1$ - L1-norm of the parameter vector,
- $\alpha$ - constant,
- $M$ - number of samples,
- $N$ - number of features ($10$ bandpower features were being used in this study).

The implementation of the LASSO used in this work was taken from the Python library *scikit-learn* and uses the coordinate descent as the algorithm to fit the coefficients (Pedregosa et al., 2011).

### A.4.1 LASSO with Least-Angle Regression

Least Absolute Shrinkage and Selection Operator with Least-Angle Regression (LASSO-LARS) is a LASSO model implemented using the LARS algorithm rather than the coordinate descent *scikit-learn*. LARS is a regression algorithm that is similar to the forward stepwise regression (Efron et al., 2004). Although its detailed description is beyond the scope of this article, some most important features of LARS will be listed in this section. The algorithm has numerous advantages over the classical implementation of LASSO. One of the most important advantages is the numeric efficiency for high-dimensional data with a relatively small sample size. Additionally, LARS is fast in terms of computation time and has proven to be more stable. On the other hand, the LARS algorithm may be particularly sensitive to noise. Since EEG data can be considered noisy by nature, this might have a crucial impact on the effectiveness of LASSO-LARS in this study.

## A.5 Ridge Regression with Radial Kernel

Ridge Regression with Radial Kernel (KernelRidge) is a combination of a linear least squares with $L2$ norm regularization and kernel transformation (Robert, 2014). The $L2$ can be defined as presented in Eq.6.

$$L2 = \min_w \|Xw - Y\|_2^2 + \alpha \|w\|_2^2 \tag{6}$$

where:

- $X \in \mathbf{R}^{M \times N}$ - input data (bandpower features),
- $Y \in \mathbf{R}^{M}$ - target (vector of reaction times),
- $\|w\|_1$ - L2-norm of the parameter vector,
- $\alpha$ - complexity parameter that controls the amount of shrinkage,
- $M$ - number of samples,
- $N$ - number of features (10 bandpower features were being used in this study).

In this study, a Radial Basis Function (RBF) was used for kernel transformation. The RBF for a feature vector $X_f \in \mathbb{R}^M$ is defined as presented in Eq.7.

$$RBF = exp(-\gamma \|X_f - X_f'\|^2) \tag{7}$$

### A.6 Support Vector Machine with Radial Basis Function

Support Vector Machine (SVM) is a supervised learning method that can be used for classification and regression problems. The mathematical formulation of SVM for regression problems can be found below (Smola and Schölkopf, 2004).

Let's denote the total number of features by $N$ and a number of observations by $M$. Given training vectors $X_i \in \mathbb{R}^N$, i = $1, \ldots, M$ and a target vector $Y \in \mathbb{R}^M$, SVM solves the following regression problem:

$$\begin{aligned} &\min_{w,b,\zeta,\zeta^*} \frac{1}{2} w^T w + C \sum_{i=1}^{M} (\zeta_i + \zeta_i^*) \\ &Y_i - w^T \phi(X_i) - b \leq \varepsilon + \zeta_i, \\ &w^T \phi(X_i) + b - Y_i \leq \varepsilon + \zeta_i^*, \\ &\zeta_i, \zeta_i^* \geq 0, i = 1, ..., M \end{aligned} \tag{8}$$

which is dual to:

$$\min_{\alpha,\alpha^*} \frac{1}{2} (\alpha - \alpha^*)^T Q (\alpha - \alpha^*) + \varepsilon e^T (\alpha + \alpha^*) - Y^T (\alpha - \alpha^*) \tag{9}$$

subject to

$$\begin{aligned} &e^T (\alpha - \alpha^*) = 0 \\ &0 \leq \alpha_i, \alpha_i^* \leq C, i = 1, ..., M \end{aligned} \tag{10}$$

where:

- $e$ is the vector of all ones,
- $C > 0$ is the upper bound,
- $Q \in \mathbf{R}^{M \times M}$,

- $Q_{ij} \equiv K(X_i, X_j) = \phi(X_i)^T \phi(X_j)$ is the kernel function.

The decision function, with independent term $\rho$ is presented in following equation:

$$\sum_{i=1}^{M} (\alpha_i - \alpha_i^*) K(X_i, X) + \rho \quad (11)$$

SVM algorithms support multiple kernel functions for input data transformation. These functions are particularly useful when dealing with complex problems that have many more features than observations. Since this is the case for the problem targeted in this study, a SVM with a RBF kernel (SVM-RBF) was used instead of a linear SVM. The RBF for a feature vector $X_f$ is defined in Eq. 7.

## B HYPERPARAMETERS OF REGRESSION METHODS

This section presents the range of hyperparameters that were used to optimize performance of the selected machine learning algorithms. For a detailed description of each of the presented hyperparameters, please refer to the documentation of the *scikit-learn* library (Pedregosa et al., 2011).

### B.1 LASSO:

- $\epsilon = 0.001$ - length of the regularization path defined as $\frac{\alpha_{min}}{\alpha_{max}}$.
- $\alpha$ - the amount of penalization chosen based on minimizing cross-validated generalization error (method built-in to *scikit-learn* implementation).
- $tol = 0.0001$ - the tolerance for the optimization.
- Maximum number of iterations to perform was $1e6$.
- Coefficients were selected cyclically for the update every iteration.
- The interception point for the model was being calculated for the computations (i.e. data was not expected to be centered).

### B.2 LASSO-LARS

- $\epsilon = 2e - 16$ - The machine-precision regularization in the computation of the Cholesky diagonal factors.
- $\alpha$ - the amount of penalization chosen based on minimizing cross-validated generalization error (method built-in to *scikit-learn* implementation).
- $tol = 0.0001$ - the tolerance for the optimization.
- Maximum number of iteration to perform was $1e5$.
- The maximum number of points ($\alpha$) on the path used to compute the residuals in the cross-validation was $1000$.
- The interception point for the model was being calculated for the computations (i.e. data was not expected to be centered).

### B.3 KernelRidge

- Before training a subset of best features was selected.
- The criteria for feature selection was either the $F$-score or $MI$. The criteria that best suited each dataset was treated as a tuned hyperparameter.
- The number of best features that would be used was selected from the set $\{1, 2, \ldots, 30\}$.

- $\alpha \in \left\{\alpha_{min} + x\frac{\alpha_{max}-\alpha_{min}}{N_\alpha-1} | x \in \{0,1,\ldots,N_\alpha-1\}, \alpha_{min} = -1, \alpha_{max} = 10, N_\alpha = 100\right\}$ - regularization strength term in L2 norm.
- $\gamma \in \left\{\gamma_{min} + x\frac{\gamma_{max}-\gamma_{min}}{N_\gamma-1} | x \in \{0,1,\ldots,N_\gamma-1\}, \gamma_{min} = 10^{-3}, \gamma_{max} = 1, N_\gamma = 100\right\}$ - gamma parameter for the RBF.

### B.4 SVMRBF

- The same feature selection procedure as presented in B.3 was utilized.
- Shrinking was always enabled during the computations.
- $C \in \left\{C_{min} + x\frac{C_{max}-C_{min}}{N_C-1} | x \in \{0,1,\ldots,N_C-1\}, C_{min} = 10^{-3}, C_{max} = 10^3, N_C = 100\right\}$ - penalty parameter of the error term.
- $\gamma = 1/N_f$ where $N_f$ denotes the number of features - kernel coefficient for RBF.
